# Diversity and Key Organisms in the Biocrust of a Tropical Granite-Gneiss Rocky Outcrop

**DOI:** 10.3390/life15050759

**Published:** 2025-05-09

**Authors:** Mateus Fernandes Oliveira, Cleber Cunha Figueredo, Adaíses Simone Maciel-Silva

**Affiliations:** 1 Programa de Pós-Graduação em Biologia Vegetal, Instituto de Ciências Biológicas, Universidade Federal de Minas Gerais, Av. Antônio Carlos, 6627, Pampulha, Belo Horizonte 31270-901, MG, Brazil; cleberfigueredo@ufmg.br (C.C.F.); adaisesmaciel@ufmg.br (A.S.M.-S.); 2 Departamento de Botânica, Instituto de Ciências Biológicas, Universidade Federal de Minas Gerais, Av. Antônio Carlos, 6627, Pampulha, Belo Horizonte 31270-901, MG, Brazil

**Keywords:** biological soil crusts, photosynthetic communities, rocky ecosystem, tropical diversity

## Abstract

Rocky outcrops are harsh habitats that support specialized organisms and communities, including biocrusts, which play roles in soil stabilization, water retention, and nutrient cycling. Despite their importance, tropical biocrusts, particularly in granite-gneiss formations, remain underexplored. This study examines biocrust composition in a granite-gneiss outcrop in a rural landscape in Southeastern Brazil, identifying microhabitats and analyzing co-occurrence patterns and community structure. We recorded eleven bryophyte species and one diatom species, while six cyanobacteria, three charophytes, and two chlorophytes were identified at the genus level. They were found in shallow depressions, though termite mounds also served as an important microhabitat. The cyanobacterium *Scytonema* was the most prevalent taxon. The liverwort *Riccia weinionis* had the highest number of positive co-occurrences, associating with cyanobacteria and algae. Network analysis based on co-occurrence revealed that *Scytonema* and the mosses *Anomobryum conicum* and *Bryum argenteum* were the most connected taxa, crucial for ecological network stability. The moss *Bryum atenense* acted as a key intermediary, with the highest betweenness centrality—a measure of its role in linking taxa. These findings provide insights into tropical rocky outcrop biocrusts, shedding light on their composition and interactions. Furthermore, the co-occurrence patterns and key taxa connectivity uncovered provide insights into ecosystem stability and can guide ecological restoration strategies.

## 1. Introduction

Rocky outcrops are isolated geological formations shaped over millions of years by erosion [1], wherein softer materials are gradually removed, leaving behind the more resistant parent rock [2]. These formations occur on all continents and take various forms depending on the type of bedrock, including sandstone escarpments, granite inselbergs, limestone cliffs, and gneissic tors [3,4,5]. In Brazil, the most common types of outcrops are granitegneiss, quartzite–sandstone, limestone, and ironstone [6], with granite formations being the most abundant [7]. These unique formations create specialized habitats that support a diverse array of endemic and highly adapted plant species [8,9,10], despite challenging environmental conditions such as intense UV radiation, high daily temperature fluctuations, low water retention, and nutrient-poor soils [9,11,12,13].

Although rocky outcrops are primarily known for their high diversity of vascular plants and adaptations to harsh environments [8,9,10,12,14,15,16,17], they also serve as essential habitats for biocrusts [7,18,19,20]. These communities, composed of both heterotrophic and autotrophic organisms, colonize the uppermost soil layer, particularly in arid and semi-arid environments [21,22]. Biocrust-forming organisms include bacteria, cyanobacteria, algae (chlorophytes, diatoms, and charophytes), bryophytes (mosses and liverworts), free-living fungi, and lichens [21,22]. Additionally, they harbor a rich diversity of microfauna, such as bacterivorous and predatory protozoans, nematodes, tardigrades, rotifers, mollusks, and arthropods, particularly mites and collembolans [23].

Similar to vascular plants from rocky outcrops, the components of biocrusts have developed a range of strategies to cope with extreme environmental conditions [24]. For instance, many cyanobacteria produce protective sheaths containing UV-absorbing compounds [25], while lichens synthesize pigments such as zeaxanthin to mitigate light stress [26]. Bryophytes, particularly mosses, develop specialized leaf structures such as hyaline hairpoints, lamellae, papillae, and alar cells to enhance water retention, while liverworts feature thickened cell walls, wall ornamentations, and dense rhizoidal mats to increase water uptake and escape dry seasons by shortening their life cycles [27,28,29,30]. Furthermore, many cyanobacteria, algae, lichens, and bryophytes show high desiccation tolerance and can survive being almost completely dried out for long periods [31,32,33].

In addition to their remarkable adaptations, biocrusts perform crucial ecological functions, including carbon [34] and nitrogen fixation [35,36], soil stabilization [37], and water retention [38], making them key components of outcrop ecosystems. However, most research on their biodiversity and functional roles has focused on arid and semi-arid regions [21], with tropical ecosystems remaining comparatively understudied. This historical bias may explain why, despite studies on biocrusts in rocky outcrops worldwide—such as in granite formations in Antarctica [18] and limestone outcrops in China [19,20]—their diversity and distribution in tropical rocky outcrops, particularly in Brazil, are still poorly understood [39]. In Brazil, attention has largely been paid to quartzite–sandstone, limestone, and ironstone formations [7,40], while granite-gneiss outcrops remain underexplored.

Granite-gneiss outcrops are characterized by harsh environmental conditions and plant communities adapted to a mosaic of microhabitats [41]. These formations often feature shallow depressions with thin layers of substrate that support specialized vegetation, creating unique ecological niches [41] that may be highly favorable for biocrust establishment. Biocrusts, which thrive in water-limited environments with minimal vascular plant competition [20], are commonly found colonizing exposed soil surfaces across various types of rocky outcrops [7]. Therefore, granite-gneiss outcrops represent promising microhabitats for biocrust development and diversity yet remain critically understudied in tropical regions.

The biodiversity of Brazilian granite-gneiss outcrops is increasingly threatened by human activities, including quarrying, mining, grazing, goat herding, fires, biological invasions, urban expansion, and the unsustainable extraction of ornamental plant species [42,43,44]. These outcrops are often located in regions where 75% of the Brazilian population resides [45], particularly in areas where urban development and agricultural activities are rapidly expanding over natural cover [46,47]. As a result, conflicts between conservation efforts and human activities are becoming increasingly pronounced. Moreover, the lack of protected areas for granite-gneiss outcrops [7] highlights the urgent need for comprehensive inventories of these formations, particularly those located on rural landscapes. Surveys of species are essential, not only for advancing ecological, genetic, biogeographic, and evolutionary research but also for informing policies aimed at conserving Brazil’s rocky outcrop ecosystems [48].

Despite the ecological importance of biocrusts, our understanding of their diversity, structures, and interactions in tropical granite-gneiss outcrops remains limited. Within this contextual framework, we conducted a study on biocrusts in a granite-gneiss rocky outcrop situated in a rural landscape in Southeastern Brazil. To enhance our understanding of the biocrust communities associated with these formations, we aimed to (I) determine the microhabitats where biocrusts develop at the study site; (II) assess the taxonomic diversity of biocrust components, focusing on bryophytes, cyanobacteria, and algae; (III) analyze co-occurrence patterns among taxa to infer potential ecological interactions; and (IV) investigate the structure and dynamics of species interactions within the biocrust community through network analysis, identifying key species that play central roles in shaping ecological relationships. By focusing on understudied tropical biocrusts and integrating ecological network analysis, we seek to fill key gaps in research on tropical biocrust biodiversity and function.

## 2. Materials and Methods

### 2.1. Study Area

**Figure 1:**
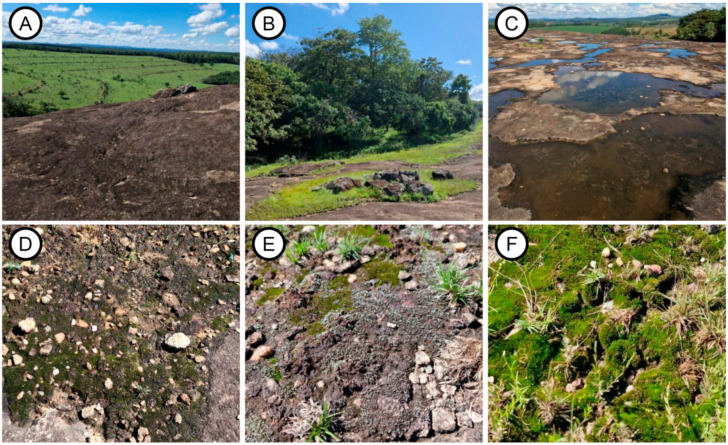
Granite-gneiss outcrop within a rural landscape in Southeastern Brazil and the associated biocrusts: (**A**) top of the outcrop, with a view of the surrounding pasture; (**B**) semi-deciduous forest present on the outcrop; (**C**) temporary ponds on the summit of the outcrop; (**D**) biocrusts dominated by cyanobacteria; (**E**) biocrusts with various species of mosses; and (**F**) biocrust dominated by a single moss species.

Our research was conducted on a granite-gneiss rocky outcrop located on a private property named Fazenda Pedra Grande in the Bocaina district, municipality of Cláudio, Minas Gerais, Brazil (20°20′25″ S, 44°52′02″ W). The outcrop is bordered by two distinct landscapes: on one side, a steep cliff faces extensive rural landscapes predominantly used for maize and soybean cultivation, as well as grazing land for livestock (Figure 1A), while the other side is surrounded by a semi-deciduous forest (Figure 1B). Mean annual minimum and maximum temperatures are 22.0 °C and 23.5 °C, respectively, and the average annual rainfall is 1411.6 mm (based on the most recent data provided by A564 (portal.inmet.gov.br)). During the rainy season, several temporary ponds can be found on the top of the outcrop (Figure 1C). The study site is located at the ecotone between the Atlantic Forest and the Cerrado, comprising a rocky outcrop with an area of approximately 170,000 m^2^. There is a notable abundance of biocrust components within the vegetation at the studied site (Figure 1D–F).

### 2.2. Biocrust Survey

**Figure 2:**
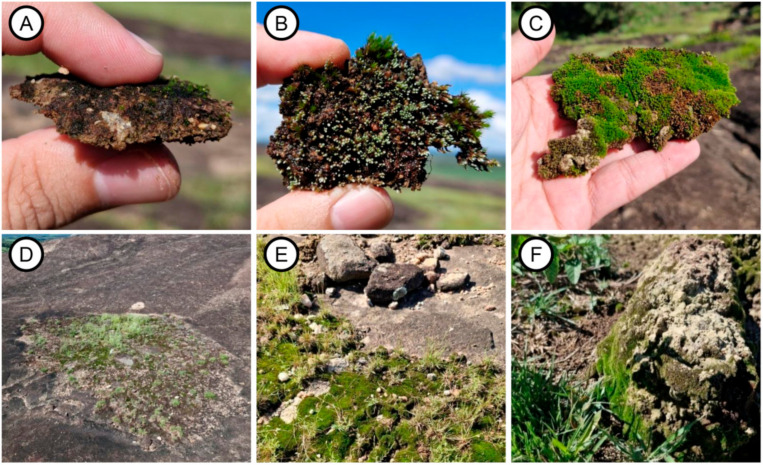
Biocrusts from the studied granite-gneiss rocky outcrop and the microhabitats where they can be found: (**A**) close-up of a biocrust dominated by cyanobacteria; (**B**) biocrust containing various components; (**C**) moss-dominated biocrust, incorporating the underlying soil; (**D**) shallow depression microhabitat; (**E**) biocrusts growing over a shallow depression; and (**F**) termite mounds atop shallow depressions.

Sampling of biocrusts was carried out on 31 December 2024, using the random walk method, which involves unrestricted movement along trails [49]. Thus, we walked along the trails of the outcrop searching for morphologically distinct samples, aiming to collect the greatest variety of species possible. Samples, each measuring approximately 100 cm^2^ and spaced at least 1 m apart, were collected from the topsoil by gently inserting a spatula into the substrate. The samples typically formed a cohesive structure, with the surface layer consisting of cryptogamic organisms, while the underlying soil was aggregated and retained beneath this upper layer (Figure 2A–C). The samples were subsequently placed in small paper bags, and the microhabitats from which they were collected were recorded.

We sampled only two microhabitats hosting biocrusts: shallow depressions (Figure 2D,E) and termite mounds (Figure 2F). These microhabitats were selected based on a study conducted on granite-gneiss formations in Southeastern Brazil [41] and another study on different outcrop formations with documented microhabitats supporting biocrusts [7]. Shallow crevices, ranging from 0.8 to 5 m in radius, were the predominant microhabitat, with biocrusts mainly developing along their edges (Figure 2E). Termite mounds, though located within crevices, were considered a distinct microhabitat, according to the classification used in previous studies [7]. They were relatively scarce, did not exceed 60 cm in height, and had low biocrust diversity.

In the laboratory, a decision tree approach [22] was applied to confirm that the collected samples represented biocrusts. Each sample was first examined under a stereomicroscope and then under a light microscope until all visible components were documented, with a focus on identifying bryophytes, cyanobacteria, and algae. In samples with cyanobacterial mats, a portion of the material was carefully removed using tweezers, and a slide was prepared for microscopic observation. For samples containing bryophytes, a slide was prepared to examine plant characteristics, while epiphytic algae and cyanobacteria were also identified. Additionally, for each sample, soil aliquots (5 mL each) from the immediate surface under the biocrust layer were diluted in 10 mL of deionized water. Using a pipette, three drops of the suspension were placed on a slide and carefully examined for the presence of algae and cyanobacteria.

All bryophytes, including mosses and liverworts, were identified up to the species level using appropriate taxonomic references [50,51,52,53,54]. Cyanobacteria and algae were identified up to the genus level, using specialized sources [55,56,57,58,59]. We opted for this less detailed identification due to the limited literature available on identifying algae and cyanobacteria in biocrusts, particularly in tropical regions [59]. However, each genus identified in the samples appeared to correspond to a single species of these microorganisms. Voucher specimens were deposited in the BHCB herbarium at the Instituto de Ciências Biológicas, Universidade Federal de Minas Gerais (voucher numbers 223811 to 223848).

### 2.3. Statistical Analysis

We analyzed taxon co-occurrence patterns in biocrusts using Veech’s method [60], implemented through the ‘cooccur’ package [61] in R 4.0.2 software [62]. To accomplish this, we constructed a species coincidence matrix based on the presence or absence of taxa in each biocrust sample from the studied rocky outcrop. This probabilistic approach excluded from the analysis any co-occurrences expected to be less than 1, thereby filtering out species pairs with a probability of sharing fewer than one sample. The probabilistic model developed by Veech [60] applies combinatorial methods to assess whether the observed frequency of co-occurrence is significantly greater than expected (positive co-occurrence), significantly lower than expected (negative co-occurrence), or not significantly different from expectations (random co-occurrence). Following this filtering process, only 82 out of 253 possible species pair combinations were analyzed.

In ecosystems, species form intricate interaction networks that shape community dynamics and ecological processes [63]. These networks, where species act as nodes connected by their relationships, define ecological interactions and drive ecosystem functions [64]. While microbial community networks in biocrusts have been widely studied [65,66,67,68], other biocrust components, such as bryophytes, remain underexplored in terms of their roles within these communities. Thus, to better understand the structure and dynamics of taxon interactions within the studied biocrust community, a biological co-occurrence network was constructed using the ‘igraph’ package [69] in R 4.0.2 [62]. This analysis focused on key parameters such as taxon connectivity, betweenness centrality, network density, community structure, community detection, and modularity.

Taxon connectivity was evaluated through degree (degree function), which measures the number of direct connections (edges) each taxon (node) has within the network [69]. Their taxa’s role as intermediaries was assessed using betweenness centrality (betweenness function), which quantifies how often a taxon acts as a bridge between other taxa in a network [69]. To evaluate overall network connectivity, network density (edge_density function) was calculated, representing the proportion of observed connections relative to the total possible connections in the network [69]. Community structure was analyzed using modularity (modularity function), which detects groups of taxa that co-occur more frequently with each other than with the rest of the network [69]. Community detection (clusters function) was performed to determine the number of taxon groups (or modules) containing more than two taxa, providing insight into how taxa are organized within the network [69]. Network modularity was assessed through a permutation test, where communities were randomly shuffled 1000 times, and modularity was calculated for each permutation. The *p*-value was determined based on the proportion of permutations where modularity was greater than or equal to the observed modularity [69].

## 3. Results

### 3.1. Biocrust Diversity in Granite-Gneiss Microhabitats

We collected 38 biocrust samples, each of which often contained multiple organisms. In total, we analyzed 168 specimens, representing six phyla (Cyanobacteria, Chlorophyta, Charophyta, Bacillariophyta, Bryophyta, and Marchantiophyta), 17 families, and 20 genera (Table 1). Cyanobacteria was the most frequently recorded phylum, with 70 occurrences, followed by Bryophyta, with 61 occurrences. Bacillariophyta was the least common, with only four records. In terms of taxonomic diversity, Bryophyta exhibited the greatest richness, comprising nine moss species distributed across four families, six genera, and nine species (Table 1).

**Figure 3:**
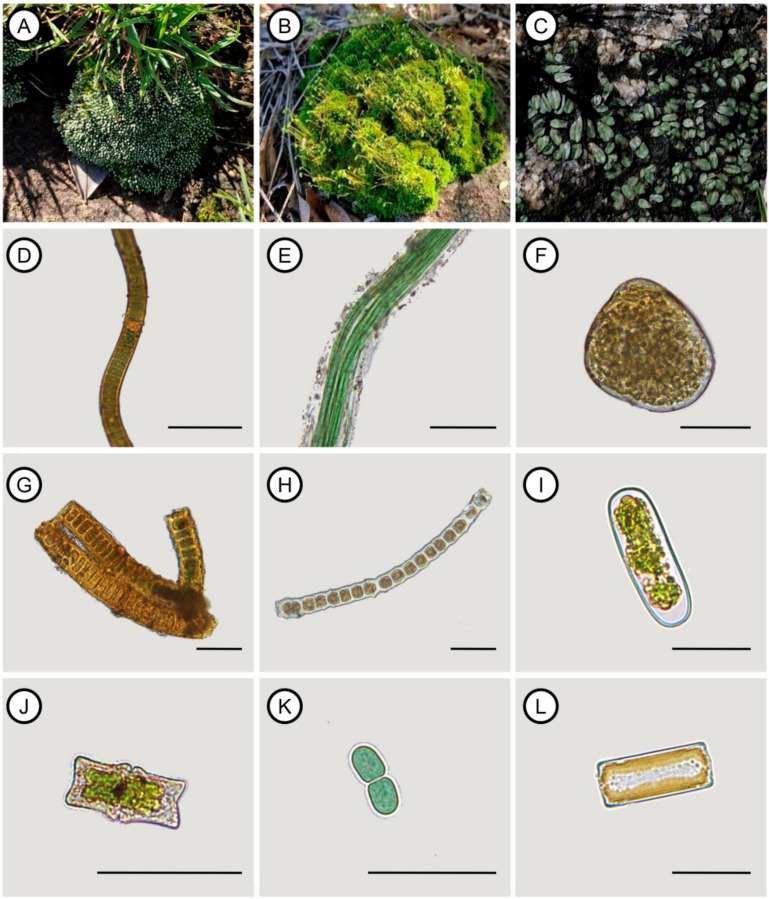
Some representatives of mosses, liverworts, cyanobacteria, and algae identified within the biocrusts of the studied granite-gneiss outcrop: (**A**) the silver moss *Bryum argenteum*; (**B**) *Bryum atenense* on a termite mound with abundant sporophytes; (**C**) *Riccia weinionis*, a complex thallose liverwort; (**D**) *Scytonema* sp., a nitrogen-fixing filamentous cyanobacterium; (**E**) *Microcoleus* sp., the most common cyanobacterium in biocrusts; (**F**) *Nostoc* sp., forming mucilaginous colonies; (**G**) *Stigonema* sp., a pseudobranching cyanobacterium; (**H**) the filamentous algae *Zygogonium* sp.; (**I**) *Actinotaenium* sp., a basal desmid; (**J**) *Euastrum* sp., with distinct semi-cell symmetry; (**K**) *Cyanothece* sp., a unicellular cyanobacterium; and (**L**) girdle view of *P. borealis*. The scale bars correspond to 50 µm.

The mosses *Bryum argenteum* Hedw. (Figure 3A) and *Bryum atenense* Williams (Figure 3B) were the most abundant among bryophytes. In contrast, some moss species were recorded only once, such as *Fabronia ciliaris* (Brid.) Brid. The thallose liverwort *Riccia weinionis* Steph. (Figure 3C) was also highly abundant in the biocrusts in the study area. Among all the organisms identified, the most frequently occurring was the cyanobacterium *Scytonema* C. Agardh ex Bornet & Flahault (Figure 3D), recorded in 34 samples. Other notable cyanobacteria included *Microcoleus* Desmazières ex Gomont (Figure 3E), *Nostoc* Vaucher ex Bornet & Flahault (Figure 3F), *Stigonema* C. Agardh ex Bornet & Flahault (Figure 3G), and *Cyanothece* Komárek (Figure 3H). Both Chlorophyta and Charophyta had the same frequency in the studied biocrusts, but *Chlorococcum* Meneghini was the most frequent green algae. Charophyta included both filamentous forms, such as *Zygogonium* Kützing (Figure 3I), and unicellular representatives like *Actinotaenium* (Nägeli) Teiling (Figure 3J) and *Euastrum* Ehrenberg ex Ralfs (Figure 3K). The only recorded diatom was *Pinnularia borealis* Ehrenberg (Figure 3L).

In the studied granite-gneiss outcrop, shallow crevices were more prevalent than termite mounds, with only four samples collected from the latter microhabitat. All these samples contained the moss *B. atenense*, which co-occurred with various taxa, including *Stigonema* sp., *Chlorococcum* sp. + *Scytonema* sp., *Chlorococcum* sp., and *F. ciliaris* + *Scytonema* sp. Notably, *F. ciliaris* was the only species exclusive to termite mounds. Liverworts, charophytes, diatoms, and several taxa of moss, chlorophytes, and cyanobacteria were absent from this microhabitat.

### 3.2. Co-Occurrence Interactions

**Figure 4:**
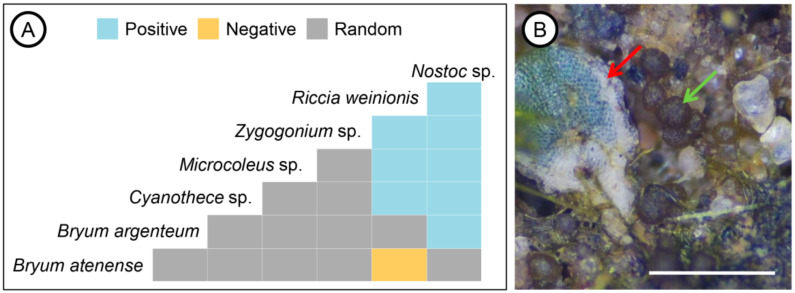
Co-occurrence patterns among the taxa from the sampled biocrusts: (**A**) observed patterns, including positive, negative, and random relationships; (**B**) *Riccia weinionis* (red arrow) and *Nostoc* sp. (green arrow) in a sample, with the cyanobacterial colonies growing on the soil surrounding the liverwort. The scale bar corresponds to 0.5 mm.

Out of the 82 pairs analyzed, the majority (73) co-occurred as expected based on the random null model, while eight pairs co-occurred more frequently than expected, and one pair co-occurred less frequently than expected (Figure 4A, Table 2). Interestingly, *R. weinionis* was included in the greatest number of significant co-occurring pairs, with positive associations with several cyanobacterial taxa, the most frequent being its co-occurrence with *Nostoc* sp. (Table 2). Indeed, in most of the samples containing *R. weinionis*, we observed several colonies of *Nostoc* sp. growing on the soil surrounding it (Figure 4B). The moss *B. argenteum* was another bryophyte showing a positive co-occurrence with *Nostoc* sp. The only negative co-occurrence relationship was observed between two bryophyte species, the moss *B. atenense* and the liverwort *R. weinionis*.

### 3.3. Community Network

**Figure 5:**
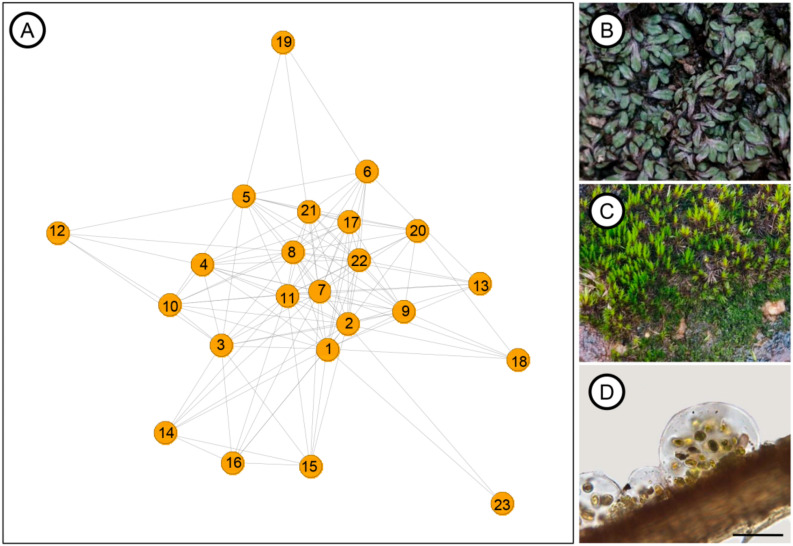
Community network of biocrusts from the studied granite-gneiss. (**A**) Community network. The numbers represent species ranked in descending order of betweenness centrality: 1—*Bryum atenense*, 2—*Anomobryum conicum*, 3—*Campylopus lamellatus*, 4—*Gloeocystis*, 5—*Pinnularia borealis*, 6—*Actinotaenium*, 7—*Scytonema*, 8—*Nostoc*, 9—*Chlorococcum*, 10—*Stigonema*, 11—*Bryum argenteum*, 12—*Euastrum*, 13—*Bryum arachnoideum*, 14—*Fissidens weirii*, 15—*Cylindrocolea rhizantha*, 16—*Gloeothece*, 17—*Zygogonium*, 18—*Dicranella lindigiana*, 19—*Bryum orthodontioides*, 20—*Cyanothece*, 21—*Riccia weinionis*, 22—*Microcoleus*, and 23—*Fabronia ciliaris*. (**B**) Example of a sample dominated by *R. weinionis*. (**C**) Sample dominated by two moss species, highlighting their co-occurrence and interaction. (**D**) *Gloeocystis* growing epiphytically on the moss *Campylopus lamellatus*. The scale bar corresponds to 50 µm.

The co-occurrence analysis enabled the construction of an interaction network among biocrust components. Firstly, the network’s modularity was calculated to be 0, indicating no strong division into subcommunities, with all species belonging to a single community. The *p*-value for the modularity permutation was 1, indicating that the observed modularity in the network was not significantly different from a random network. The network density was 0.49, suggesting moderate connectivity between taxa. These results indicate a highly co-occurring network, implying an integrated and interdependent ecological structure (Figure 5A). This was also evident during the biocrust analysis in the laboratory, where some samples were dominated solely by one bryophyte (Figure 5B), but upon closer inspection, we found a wide diversity of photosynthetic microorganisms associated with them. In some samples, for instance, more than two moss species were found growing on a single mat (Figure 5C), with algae and cyanobacteria observed growing epiphytically on these mosses (Figure 5D), though not exclusively associated with them.

The degree of co-occurrence of the taxa in the network revealed that *Scytonema* sp., *Anomobryum conicum* (Hornsch.) Broth., *B. argenteum*, *B. atenense*, and *Nostoc* sp. were the most connected species (Table 3), highlighting their central role in maintaining structural integrity and ecological interactions within the biocrust community. In contrast, *F. ciliaris*, with the lowest degree, exhibited fewer interactions, suggesting a more peripheral role in the network (Table 3). Regarding betweenness centrality, which measures the mediation between species, *B. atenense* had the highest betweenness centrality, followed by *A. conicum*, *Campylopus lamellatus* Mont., *Gloeocystis* sp., and *Pinnularia borealis* (Table 3), underscoring their importance as key intermediaries in the network. The lowest betweenness centrality in the network was observed for *F. ciliaris*, followed by *Microcoleus* sp. and *R. weinionis*, indicating that these taxa play a more peripheral role in mediating interactions between other taxa in the co-occurrence network (Table 3).

## 4. Discussion

### 4.1. Biocrusts of Brazilian Rocky Outcrops: A Comparative Perspective

Our findings highlight the hidden diversity within biocrusts, suggesting that further studies may uncover even greater taxonomic richness. We report, for the first time, that the green algae genus *Actinotaenium* is a component of biocrusts, a genus that was not included in the most recent review [59]. The bryophytes *A. conicum*, *D. lindigiana*, *F. ciliaris*, *F. weirii*, and *R. weinionis* are also newly reported components of biocrusts, having not been confirmed in biocrusts from any other environment worldwide [70]. Although research on biocrusts in Brazil has predominantly focused on cyanobacteria [71,72,73,74], we report, for the first time, that the genus *Cyanothece* is a component of biocrusts in Brazilian ecosystems, although this genus is already recognized in biocrusts of drylands [75,76]. Emerging studies have started to document a broader diversity of biocrust components [7,40,77,78,79], but this study contributes to expanding the understanding of biocrust biodiversity by revealing new records of species previously undocumented in Brazilian biocrust communities, such as *Chlorococcum*, *Euastrum*, and *P. borealis*. Although we only used microscopy, we were still able to obtain these relevant records, and more advanced methods for cyanobacteria and algae identification could reveal even greater diversity.

Some of the biocrust organisms recorded in the granite-gneiss outcrop, such as the mosses *B. atenense* and *B. argenteum*, as well as cyanobacterial genera like *Microcoleus* and *Scytonema*, are shared across various Brazilian outcrops [7,40]. In contrast, taxa such as *Chlorococcum*, *Euastrum*, *P. borealis*, *F. weirii*, and *R. weinionis* are, to date, unique to the granite-gneiss site. This suggests that, although some taxa are widespread, each substrate type could support distinct species, a potentiality shaped by factors such as soil chemistry, moisture availability, and microclimatic conditions [80]. These results highlight the role of substrates in structuring biocrust communities. However, little is known about how soil composition influences these communities, and addressing this gap should be a priority for future ecological studies on biocrusts.

In bryophyte-dominated biocrusts, the moss families Pottiaceae and Bryaceae, along with the liverwort family Ricciaceae, are typically dominant [70], but with some differences across rocky outcrops. In limestone formations, for example, Pottiaceae and Bryaceae dominate, with liverworts being absent [7], while ironstone and quartzite–sandstone formations host both leafy liverworts and mosses from the Bryaceae and Dicranaceae families [7,40]. In our study, no Pottiaceae were found, and the biocrusts were dominated by Bryaceae, Dicranaceae, and Ricciaceae. The absence of Pottiaceae may be attributed to the acidic nature of granite-gneiss [81], as this family is often linked to calcareous soils [82,83]. The presence of Ricciaceae suggests that temporary water availability (i.e., temporary ponds) influences biocrust composition, as many *Riccia* species thrive in areas where water accumulates after rainfall [84,85].

Filamentous cyanobacteria, such as *Microcoleus* and *Scytonema*, are among the most frequently recorded taxa in biocrusts worldwide [59], including in Brazil [39]. Both genera show very relevant ecological functions, since *Microcoleus* enhances soil cohesion and water retention through extracellular polymeric substances [86,87,88], while *Scytonema* contributes to nitrogen fixation, enriching the substrate and supporting other biocrust organisms [89,90]. Their presence across multiple outcrop types—granite-gneiss, quartzite–sandstone, ironstone, and limestone [7]—highlights their role in soil stabilization and nutrient cycling. In contrast, unicellular algae and cyanobacteria are rarely documented in Brazilian biocrusts [39].

Microhabitats within the granite-gneiss outcrop share similarities with those in quartzite–sandstone and ironstone formations while also being notably distinct from those in limestone formations [7]. In fact, neither shallow depressions nor termite mounds are found in limestone formations [7]. In contrast, both quartzite–sandstone and ironstone outcrops present soil islands, which closely resemble shallow depressions. These soil islands are subject to intense sunlight and harsh environmental conditions, making them areas where moss species like *C. lamellatus*, *B. atenense*, and *B. argenteum* are frequently found [7,91,92]. These same species were found in shallow depressions within the granite-gneiss outcrop, showcasing their ability to thrive in stressful environments. Notable adaptations include the hyaline hairpoint and leaf lamellae in *C. lamellatus* and the tightly overlapping leaves of *B. argenteum*, which aid in water absorption and retention and minimizing water loss [24,28,93]. Additionally, the most frequently observed moss species on termite mounds in these formations is *B. atenense* [7], as we observed at the studied site.

### 4.2. Insights into Biocrust Components’ Co-Occurrence

The positive association observed between bryophytes and cyanobacteria in the granite-gneiss outcrop suggests potential beneficial interactions. Many bryophytes, including mosses, hornworts, and liverworts, form symbiotic relationships with nitrogen-fixing cyanobacteria [94,95,96]. The moss–cyanobacteria association occurs where nitrogen is scarce, with N_2_-fixing cyanobacteria such as *Nostoc* growing epiphytically and providing nitrogen in exchange for carbon and sulfur [97,98]. The positive co-occurrence between *B. argenteum* moss and *Nostoc* may reflect a similar beneficial relationship, as other *Bryum* species show similar interactions [99]. In contrast, only four liverwort genera establish symbioses with cyanobacteria [95], and the *Riccia* genus found in the studied biocrusts does not appear to form such associations. However, we observed *Nostoc* growing around *R. weinionis*, which may indicate a potential interaction between these two taxa. This observation warrants further investigation to determine whether it represents a previously unreported symbiotic relationship.

All the positive co-occurrence patterns observed between pairs of cyanobacteria or between cyanobacteria and algae in the granite-gneiss outcrop biocrusts involved *Nostoc*. When present in biocrusts, *Nostoc* can retain moisture [100,101] and increase nitrogen levels in the crust [35,102], potentially influencing community composition. We suspect that these effects drive the co-occurrence of *Cyanothece* with *Nostoc*. Since *Cyanothece* is predominantly found in aquatic environments and is a nitrogen fixer [103], its presence in biocrusts could be facilitated by the microclimatic buffering provided by *Nostoc* rather than by nitrogen availability alone. The positive pattern between *Microcoleus* and *Nostoc* may be explained by their roles in the early stages of biocrust formation, where they contribute to the initial soil carbon and nitrogen inputs [35,89]. Similarly, the relationship between *Zygogonium*, an alga that initiates biocrusts [89,104], and *Nostoc* may reflect a similar early colonization stage.

Finally, the only negative co-occurrence pattern observed was between two bryophytes. Liverworts, known for producing specialized metabolites in oil bodies [105,106], play a role in allelopathy, inhibiting the growth of neighboring organisms [107]. This ecological interaction allows one species to release chemical compounds that affect (negatively or sometimes positively) the growth or survival of others, securing more resources [108,109]. The presence of allelopathic compounds in *R. weinionis* may explain the negative pattern observed with *B. atenense*. However, bryophyte species coexistence does not appear to be related to inter-specific competition [110], suggesting that other factors, such as differences in environmental preferences and niche requirements, could also influence species distribution and co-occurrence [111]. Future studies should further investigate these two possibilities—whether chemical interactions, like allelopathy, or ecological niche differentiation better explain the observed pattern, considering the various environmental variables that could be shaping this dynamic.

### 4.3. Lessons from Community Network Dynamics

In the biocrusts of the granite-gneiss outcrop, cyanobacteria were the most prevalent group; however, mosses such as *B. atenense*, *A. conicum*, and *C. lamellatus* played a pivotal role in shaping the community structure. Mosses contribute to the surface roughness of biocrusts, providing an ideal substrate for the establishment of algae and cyanobacteria [112], and there are numerous records of these organisms growing on the leaves of mosses within biocrusts [7]—a phenomenon also observed in this study. Furthermore, certain algae, like *Chlorococcum*, are commonly associated with bryophytes due to their ability to retain water [89]. Despite these valuable insights, further research is necessary to fully comprehend the complex interactions within biocrust communities, particularly the role of bryophytes in influencing ecosystem functions and dynamics.

Regarding network modularity, a value of zero was observed, indicating the absence of a clear subcommunity structure. This suggests thatrather than forming distinct clusters, species interactions in the studied ecosystem may be randomly distributed [69,113]. The permutation test *p*-value of 1 further supports this, showing no significant difference from a random network. The lack of modularity points to random species interactions [113], emphasizing the need for further exploration of these ecological dynamics in biocrusts within tropical granite-gneiss outcrops.

Network analyses revealed key species within the community while also identifying critical species that could aid in the restoration of granite outcrops, which face significant anthropogenic threats [42,43,44]. Biocrusts perform essential ecosystem functions [21], and their inoculation in various contexts, such as areas impacted by mining [114,115,116] and desertification [117], has demonstrated environmental benefits. In fact, their application in restoration programs is wellestablished [118,119,120], yet it remains largely unexplored in regard to tropical rocky outcrops. Notably, recent studies suggest that inoculating terricolous mosses in Brazilian quartzite–sandstone outcrops can promote biocrust formation and support vegetation succession around rocks within a few years [40]. However, fundamental steps, such as selecting suitable species for these restoration practices, have yet to be taken.

From this perspective, the concept of interaction networks becomes particularly valuable. In our analysis, species such as *B. atenense* and *C. lamellatus* emerged as central nodes, exhibiting high connectivity within the network. These species are known to possess life-history traits associated with rapid reproduction and resilience in harsh environments [50,54], suggesting that they may have potential for use in future restoration trials. Similarly, cyanobacteria such as *Nostoc* and *Scytonema* showed strong associations with other taxa, likely due to their capacity for nitrogen fixation and soil stabilization [86,87,88,89,90]. While our results do not directly test restoration outcomes, they point to a promising avenue for future research: exploring whether the introduction of these highly connected species could enhance ecological benefits, promote the recruitment of other organisms, and contribute to greater biodiversity and ecosystem stability in biocrust restoration efforts.

## 5. Conclusions

Although this study investigates biocrust diversity at a single site and is based on a limited number of samples, it contributes valuable insights into the diversity of biocrusts on tropical rocky outcrops. It provides new species records and highlights the importance of studying community dynamics in these unique ecosystems. For the first time, we have identified diatoms within Brazilian biocrusts and highlighted the presence of bryophytes, which are often overlooked. The observed positive co-occurrence between bryophytes and cyanobacteria suggests beneficial ecological interactions, with mosses acquiring nitrogen and cyanobacteria benefiting from carbon and sulfur. On the other hand, the negative co-occurrence between liverworts and mosses raises the possibility of allelopathic effects or ecological niche differentiation. The identification of central species within biocrust networks enhances our understanding of community structure and dynamics, offering valuable insights forrestoration strategies. Incorporating these key species into ecological restoration projects can improve soil aggregation, enhance carbon and nitrogen fixation, and foster greater biodiversity. We also acknowledge that the limited sampling efforts and lack of spatial replication may affect the interpretation of ecological patterns. Therefore, we emphasize the importance of conducting further studies on other granite-gneiss outcrops using a larger number of samples to strengthen and expand upon the findings presented here.

## Figures and Tables

**Table 1:** Taxa recorded in biocrusts from the studied granite-gneiss outcrop and their specimen counts (frequency).

Phylum	Family	Taxa	Freq.
Cyanobacteria	Cyanothecaceae	*Cyanothece* Komárek	05
	Microcystaceae	*Gloeothece* Nägeli	03
	Microcoleaceae	*Microcoleus* Desmazières ex Gomont	11
	Nostocaceae	*Nostoc* Vaucher ex Bornet &Flahault	11
	Scytonemataceae	*Scytonema* C.Agardh ex Bornet &Flahault	34
	Stigonemataceae	*Stigonema* C.Agardh ex Bornet &Flahault	06
		Total	70
Chlorophyta	Chlorococcaceae	*Chlorococcum* Meneghini	07
	Radiococcaceae	*Gloeocystis* Nägeli	04
		Total	11
Charophyta	Desmidiaceae	*Actinotaenium* (Nägeli) Teiling	04
		*Euastrum* Ehrenberg ex Ralfs	01
	Zygnemataceae	*Zygogonium* Kützing	06
		Total	11
Bacillariophyta	Pinnulariaceae	*Pinnularia borealis* Ehrenberg	04
		Total	04
Bryophyta	Bryaceae	*Anomobryum conicum* (Hornsch.) Broth.	14
		*Bryum arachnoideum* Müll. Hal.	03
		*Bryum argenteum* Hedw.	17
		*Bryum atenense* Williams	15
		*Bryum orthodontioides* Müll.Hal.	01
	Dicranaceae	*Campylopus lamellatus* Mont.	07
		*Dicranella lindigiana* (Hampe) Mit.	02
	Fabroniaceae	*Fabronia ciliaris* (Brid.) Brid.	01
	Fissidentaceae	*Fissidens weirii* Mitt.	01
		Total	61
Marchantiophyta	Cephaloziellaceae	*Cylindrocolea rhizantha* (Mont.) R.M.Schust.	01
	Ricciaceae	*Riccia weinionis* Steph.	08
		Total	09

**Table 2:** Co-occurrence tests between biocrust taxa. The tests were conducted using the p_lt and p_gt values, which represent the probability of observing a co-occurrence smaller or larger than expected by chance. The p_lt values below 0.05 indicate a significantly smaller co-occurrence, while the p_gt values below 0.05 indicate a significantly larger co-occurrence, than expected by chance. Significant values are highlighted in bold.

Taxa Pair	p_lt	p_gt
*Riccia weinionis* and *Cyanothece*	0.999	**0.004**
*Riccia weinionis* and *Microcoleus*	0.997	**0.031**
*Riccia weinionis* and *Nostoc*	1.000	**<0.001**
*Riccia weinionis* and *Zygogonium*	0.999	**0.012**
*Riccia weinionis* and *Bryum atenense*	**0.007**	1.000
*Bryum argenteum* and *Nostoc*	0.995	**0.031**
*Nostoc* and *Zygogonium*	0.995	**0.047**
*Nostoc* and *Cyanothece*	0.999	**0.019**
*Nostoc* and *Microcoleus*	0.995	**0.036**

**Table 3:** Degree and betweenness centrality values for the components of the biocrust community.

Phylum	Taxa	Degree	Betweenness Centrality
Cyanobacteria	*Cyanothece*	10	2.508
	*Gloeothece*	7	5.799
	*Microcoleus*	13	1.537
	*Nostoc*	16	11.290
	*Scytonema*	22	12.181
	*Stigonema*	10	7.012
Chlorophyta	*Chlorococcum*	12	10.260
	*Gloeocystis*	10	15.954
Charophyta	*Actinotaenium*	10	14.892
	*Euastrum*	5	6.082
	*Zygogonium*	12	5.484
Bacillariophyta	*Pinnularia borealis*	13	15.843
	*Anomobryum conicum*	18	20.988
Bryophyta	*Bryum arachnoideum*	7	5.839
	*Bryum argenteum*	18	6.684
	*Bryum atenense*	17	36.327
	*Bryum orthodontioides*	3	3.386
	*Campylopus lamellatus*	13	16.104
	*Dicranella lindigiana*	5	3.512
	*Fabronia ciliaris*	2	0.698
	*Fissidens weirii*	7	5.799
Marchantiophyta	*Cylindrocolea rhizantha*	7	5.799
	*Riccia weinionis*	11	1.737

## Data Availability

All data generated or analyzed during this study are included in this article.
